# Milk Allergen Micro-Array (MAMA) for Refined Detection of Cow’s-Milk-Specific IgE Sensitization

**DOI:** 10.3390/nu15102401

**Published:** 2023-05-21

**Authors:** Victoria Garib, Daria Trifonova, Raphaela Freidl, Birgit Linhart, Thomas Schlederer, Nikolaos Douladiris, Alexander Pampura, Daria Dolotova, Tatiana Lepeshkova, Maia Gotua, Evgeniy Varlamov, Evgeny Beltyukov, Veronika Naumova, Styliani Taka, Alina Kiyamova, Stefani Katsamaki, Alexander Karaulov, Rudolf Valenta

**Affiliations:** 1 Center for Pathophysiology, Infectiology and Immunology, Institute of Pathophysiology and Allergy Research, Medical University of Vienna, 1090 Vienna, Austria; viktoriya.garib@meduniwien.ac.at (V.G.);; 2 International Center of Molecular Allergology, Ministry of Innovation Development, Tashkent 100174, Uzbekistan; 3 Laboratory of Immunopathology, Department of Clinical Immunology and Allergy, Sechenov First Moscow State Medical University, 119991 Moscow, Russia; 4 Allergy Department, 2nd Pediatric Clinic, National & Kapodistrian University of Athens, 11527 Athens, Greece; 5 Department of Allergology and Clinical Immunology, Research and Clinical Institute for Pediatrics Named after Yuri Veltischev at the Pirogov Russian National Research Medical University of the Russian Ministry of Health, 117997 Moscow, Russia; anpampura1@gmail.com (A.P.); ev4832525@gmail.com (E.V.); 6 Department of Bioinformatics, Department of Pediatric Surgery, Pirogov Russian National Research Medical University of the Russian Ministry of Health, 117997 Moscow, Russia; 7 Department of Faculty Therapy, Endocrinology, Allergology and Immunology, Ural State Medical University, 620028 Ekaterinburg, Russia; 8 Center of Allergy and Immunology, 123182 Tbilisi, Georgia; 9 NRC Institute of Immunology FMBA of Russia, 115478 Moscow, Russia; 10 Karl Landsteiner University for Health Sciences, 3500 Krems, Austria

**Keywords:** cow’s milk allergy, allergen molecules, milk allergen micro-array, peptides, anaphylaxis, milk tolerance, diagnosis, IgE sensitization, children

## Abstract

Background: Immunoglobulin-E(IgE)-mediated hypersensitivity to cow’s milk allergens is a frequent cause of severe and life-threatening anaphylactic reactions. Besides case histories and controlled food challenges, the detection of the IgE antibodies specific to cow’s milk allergens is important for the diagnosis of cow-milk-specific IgE sensitization. Cow´s milk allergen molecules provide useful information for the refined detection of cow-milk-specific IgE sensitization. Methods: A micro-array based on ImmunoCAP ISAC technology was developed and designated milk allergen micro-array (MAMA), containing a complete panel of purified natural and recombinant cow’s milk allergens (caseins, α-lactalbumin, β-lactoglobulin, bovine serum albumin-BSA and lactoferrin), recombinant BSA fragments, and α-casein-, α-lactalbumin- and β-lactoglobulin-derived synthetic peptides. Sera from 80 children with confirmed symptoms related to cow’s milk intake (without anaphylaxis: *n* = 39; anaphylaxis with a Sampson grade of 1–3: *n* = 21; and anaphylaxis with a Sampson grade of 4–5: *n* = 20) were studied. The alterations in the specific IgE levels were analyzed in a subgroup of eleven patients, i.e., five who did not and six who did acquire natural tolerance. Results: The use of MAMA allowed a component-resolved diagnosis of IgE sensitization in each of the children suffering from cow’s-milk-related anaphylaxis according to Sampson grades 1–5 requiring only 20–30 microliters of serum. IgE sensitization to caseins and casein-derived peptides was found in each of the children with Sampson grades of 4–5. Among the grade 1–3 patients, nine patients showed negative reactivity to caseins but showed IgE reactivity to alpha-lactalbumin (*n* = 7) or beta-lactoglobulin (*n* = 2). For certain children, an IgE sensitization to cryptic peptide epitopes without detectable allergen-specific IgE was found. Twenty-four children with cow-milk-specific anaphylaxis showed additional IgE sensitizations to BSA, but they were all sensitized to either caseins, alpha-lactalbumin, or beta-lactoglobulin. A total of 17 of the 39 children without anaphylaxis lacked specific IgE reactivity to any of the tested components. The children developing tolerance showed a reduction in allergen and/or peptide-specific IgE levels, whereas those remaining sensitive did not. Conclusions: The use of MAMA allows for the detection, using only a few microliters of serum, of IgE sensitization to multiple cow’s milk allergens and allergen-derived peptides in cow-milk-allergic children with cow-milk-related anaphylaxis.

## 1. Introduction

An intolerance to cow’s milk is particularly common. In most cases, it is due to lactose intolerance and can be avoided by the consumption of lactose-free milk products. Immunoglobulin-E-(IgE)-mediated cow’s milk allergy (CMA) is more rare and affects approximately 3% of the population [1,2]. CMA occurs very early in childhood when children are exposed to cow’s milk (CM) in their diet. Despite the relatively low number of sensitized patients, CMA is especially important to diagnose because it can cause severe and life-threatening anaphylaxis, which may lead to death upon the consumption of CM [3,4]. The cornerstone for a diagnosis of IgE-mediated CMA is a well-documented case history documenting immediate occurrences of allergic symptoms that can be unambiguously attributed to the consumption of CM [5,6]. Whenever possible, it is important to confirm CMA via a controlled food challenge in a safe clinical setting [4,7]. IgE sensitization to CM allergens should be confirmed by the detection of allergen-specific IgE antibodies with serological measurements. Furthermore, skin testing and basophil activation tests are useful for demonstrating the allergenic activity of CM in a given patient; however, the basophil activation test is mainly used for research [4,8,9].

Traditionally, the serological detection of CM-allergen-specific IgE antibodies is performed with the use of CM allergen extracts, of which some are able to help quantify CM-allergen-specific IgE levels [10]. Interestingly, the quantification of CM-allergen-specific IgE antibodies has turned out to be useful for the prediction of the severity of CM-related allergic symptoms; however, the cut-off levels are not sharp and have been found to vary in different populations [10,11]. Similar to many other allergen sources, it has become possible to purify the individual allergen molecules in cow’s milk to produce them as recombinant allergen molecules and to synthesize CM-allergen-derived peptides for the mapping of the IgE, IgG and T-cell epitopes that are recognized by CM-allergic patients [11,12,13]. Purified allergen molecules and the peptides derived thereof have brought forward another era in allergy diagnosis, one which is commonly referred to as component-resolved allergy diagnosis or molecular allergy diagnosis [13,14,15,16]. Cow’s milk contains different amounts of allergen molecules that show different resistances against heating and digestion, and which, accordingly, have been suggested to be associated with very severe forms, as well as severe and mild forms of CMA. In addition, some compounds such as BSA do not seem to cause the relevant symptoms of CMA [9,15,17,18]. Individual CM allergens became available in the form of single allergen components, which allow for the measurement of IgE levels that are specific for certain allergens. In parallel to this, multiplex allergen tests have been developed, which allow for the simultaneous measurement of IgE levels for several allergens and/or allergen peptides by macro-, micro-array technology, or by bead-based assays [16,17,19,20,21,22]. In using such tests, interesting information was obtained regarding IgE reactivity profiles, as well as regarding IgE levels to certain allergens and/or allergen-derived peptides, such as biomarkers for different phenotypes, the severities of CM allergy, the outgrowth of CM allergy and the monitoring of allergen-specific immunotherapy [10,13,21,22,23,24,25]. 

In this study, we present a novel milk allergen micro-array (MAMA) that allows one to measure, with only a few microliters of serum, the IgE levels of to all known CM allergens and certain CM-allergen-derived peptides. In addition, MAMA was also used for the high-resolution mapping of allergen- and peptide-specific IgE reactivity profiles in a cohort of clinically well-defined CM-allergic children. Our study not only provides a multiplex assay that allows a hitherto unmatched detailed survey of CM allergen- and peptide-specific IgE reactivity profiles, but it also appears to be useful for the reliable detection of CM-allergen-specific IgE reactivity in patients with anaphylactic reactions to CM, as well as for monitoring the development of patient tolerance following an elimination diet.

## 2. Materials and Methods

### 2.1. Cow-Milk-Allergic Patients, Sera and PBMC Samples 

Children suffering from CMA (Table 1) were observed in the Allergy Department, 2nd Pediatric Clinic University of Athens, Greece; in the Center of Allergy and Immunology, Tbilisi, Georgia; in the Department of Allergology and Clinical Immunology, Research and Clinical Institute for Pediatrics named after Yuri Veltischev at the Pirogov Russian National Research Medical University, Moscow, Russia; and in the Department of Faculty Therapy, Endocrinology, Allergology and Immunology, Ural State Medical University, Ekaterinburg, Russia. For patients with anaphylactic reactions to CM, the diagnosis of CMA was based on specific criteria [26], including the presence of clinical symptoms of an immediate type of CMA (which could only be unambiguously attributed to CM) and a clear-cut history of anaphylaxis following milk consumption, in accordance with published guidelines [27]. Skin prick testing with CM allergen extracts and/or the measurement of IgE levels specific for CM allergen extracts obtained with ImmunoCAP (Thermo Fisher Scientific, Uppsala, Sweden) was performed in the study population (Table 1).

The CM-related allergic symptoms of patients were ranked as non-anaphylactic and anaphylactic. Anaphylactic reactions were graded as grades 1–5 according to Sampson [28]. The majority of CM-allergic patients (i.e., 52/80) were also sensitized to other food allergen sources such as egg, peanut, wheat, nuts, soy, cereals, fish and caviar.

A subset of 11 children (Table 2) were subjected to a milk elimination diet, (#1, 11, 25, 30, 32, 36, 38, 70) or a milk modification diet (i.e., they received only baked milk) (#65–67).

Episodes of anaphylaxis due to accidental intake of cow’s milk were recorded by the treating physicians.

The development of clinical tolerance in the form of baked, fermented, or whole milk when re-introduced into the diet under controlled conditions was monitored also by the treating physicians. No controlled challenges were performed in the clinics.

Written informed consent was obtained from the parents or legal guardians of the children in order to obtain blood samples and immunological analysis (these were also approved by the corresponding local ethics committees in Greece, Georgia and Russia). The analysis of the pseudonymized samples with MAMA was conducted with approval from the Ethics Committee of the Medical University of Vienna, Austria (EK1641/2014).

### 2.2. ImmunoCAP ISAC Measurements

Allergen-specific IgE antibodies were measured in certain serum samples with ImmunoCAP ISAC micro-arrays containing 112 allergens, and this was conducted according to the manufacturer’s recommendations (Thermo Fisher, Uppsala, Sweden) with permission from the Ethics Committee of the Medical University of Vienna (EK1641/2014).

### 2.3. Milk Allergen Micro-Array (MAMA)

The natural and recombinant milk allergens, recombinant BSA fragments and milk-allergen-derived synthetic peptides are summarized and characterized in Table S1 in the Supplementary Materials. The spotting of proteins and peptides was performed as described in [29]. However, in more detail, the glass slides containing six microarrays were surrounded by an epoxy frame (Paul Marienfeld GmbH & Co. KG, Lauda-Königshofen, Germany) and were coated with an amine-reactive complex organic polymer, MCP-2 (Lucidant Polymers, Sunnyvale, CA, USA). This surface was meant to facilitate the immobilization of the proteins and peptides. The spotting conditions, buffers and concentrations in the pilot experiments to obtain round-shaped and compact spots of comparable size were optimized for each protein/peptide. During the final printing stage, milk allergens were spotted, in triplicates, in concentrations of 0.5–1 mg/mL in phosphate buffer (75 mM Na_2_HPO_4_, pH = 8.4) with a SciFlexArrayer S12 (Scienion AG, Berlin, Germany).

IgE reactivity and IgE levels specific for micro-arrayed proteins and peptides were measured as follows: The microarrays were washed for 5 min with phosphate-buffered saline containing 0.5% Tween 20 (PBST) and were dried via centrifugation with a Sigma 2–7 centrifuge and MTP-11113 rotor (both Sigma Laborzentrifugen GmbH, Osterode am Harz, Germany). Subsequently, 35 µL of undiluted serum sample was added per array and incubated for 2 h at 22 °C. After another washing step, the bound IgE antibodies were detected as described in [30]. The slides were again washed, dried and subsequently scanned with a confocal laser scanner (Tecan, Männedorf, Switzerland).

Image analysis was performed via MAPIX microarray image acquisition and analysis software version 8.5.0 (Innopsys, Carbonne, France), and through a conversion of measured fluorescence units to ISAC standardized units (ISU), which was performed as described in [31].

### 2.4. Statistical Analyses

Given the small number of patients in the studied groups, the median and the interquartile range (Me [Q1; Q3]) were used in the description of the variable distributions. The pairwise comparison of groups was performed with a Mann–Whitney U test. Considering the problem of multiple hypothesis testing, the significance threshold was Holm-corrected to 0.000385 [32].

The graphical representation included box and whisker diagrams that demonstrated the median (a horizontal line inside the “box”), the first and third quartiles (upper and lower bounds of the “box”) and the minimum and maximum (“whiskers”). Additionally, the distribution of allergen values within the ranges of [0.3; 1), [1; 15) and ≥15 was illustrated using different color codes.

The analysis was performed using IBM SPSS Statistics 20.0 (New York, NY, USA).

The graphical representation was performed using GraphPad Prism 6 software (GraphPad Software, La Jolla, CA, USA), RStudio software 2022.11.4-20 and Microsoft Office Excel 2010. Correlations of allergen-specific IgE levels determined by MAMA and ImmunoCAP ISAC were assessed by Spearman´s rank correlation coefficient. *p* values of <0.05 were considered as significant.

## 3. Results

### 3.1. Characterization of CM-Allergic Patients

In this study, we investigated 80 subjects (28 females, 52 males, age range 0.5–12 years, median age 3.1 years) with allergic symptoms that could be clearly attributed to the consumption of CM (Table 1). In total, 60 of the children showed skin symptoms, 42 showed gastrointestinal symptoms and 41 showed systemic reactions (Table 1). Among the 41 children with systemic reactions, 21 presented reactions at grades 1–3 (according to Sampson [24]) and 20 presented grade 4–5 reactions (Table 1). Mild symptoms (skin reactions, gastrointestinal reactions, etc.) without systemic reactions were found in 39 patients (Table 1). The results from skin prick testing and from the measurements of specific IgE to CM allergen extracts were available for 46 and 41 of the 80 CM-allergic patients, respectively (Table 1).

Table 2 shows the characteristics of patients (*n* = 11) who were prescribed a CM elimination or modification diet. The duration of the prescribed diet was from 8 to 18 months. Two children suffered from CM-related anaphylaxis at grade 4–5, five from anaphylaxis grade 1–3 and four had mild symptoms (Table 2). Five of the children (#1: grade 5; #25: grade 3; #65: grade 0; #66: grade 0; and #11: grade 4) did not develop a tolerance, whereas six developed a tolerance (i.e., #36: grade 2; #32: grade 2; #38: grade 2; #67: grade: 0; #70: grade: 0; #30: grade 3) and the rest developed some form of tolerance (Table 2). The duration of the elimination diet was not associated with the development of tolerance.

### 3.2. Creation of MAMA

**Figure 1:**
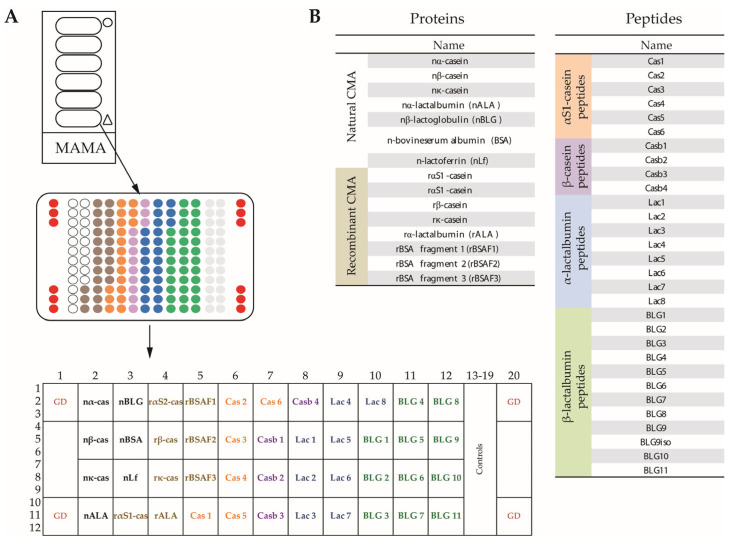
The layout and composition of the milk allergen micro-array (MAMA). (**A**) Glass slides containing six micro-arrays surrounded by an epoxy frame, magnification of one micro-array showing the order of dots (triplicates), and further magnification showing guide dots (GD), positions of cow’s milk (CM) allergens and peptides in boxes. (**B**) Lists of the natural CM allergens, recombinant CM allergens and CM-derived peptides according to Table S1.

Each of the glass slides (i.e., chips) contained six milk allergen micro-arrays (MAMAs), which were surrounded by a frame to avoid an overflow of serum (Figure 1A). The MAMA comprised triplicate spots of each allergen and allergen-derived peptides together with four guide dot triplicates (red), as is indicated in Figure 1A. The MAMA contained purified natural CM allergens, recombinant CM allergens, recombinant BSA fragments and allergen-derived peptides, as is indicated in Figure 1A,B. The sources and characteristics of the proteins and peptides, together with the key references [9,33,34] describing the recombinant proteins and peptides are summarized in Table S1. The MAMA allowed the testing of antibody reactivity against 44 components with a volume of approximately 30 microliters of serum, plasma or other body fluids. Hence, it is especially suitable for the analysis of samples from children and when it is difficult to obtain larger volumes of test substances.

### 3.3. MAMA, but Not ImmunoCAP, or Skin Testing with CM Extracts Identifies all Patients with CMA According to a Sampson Score of 1–5

**Figure 2:**
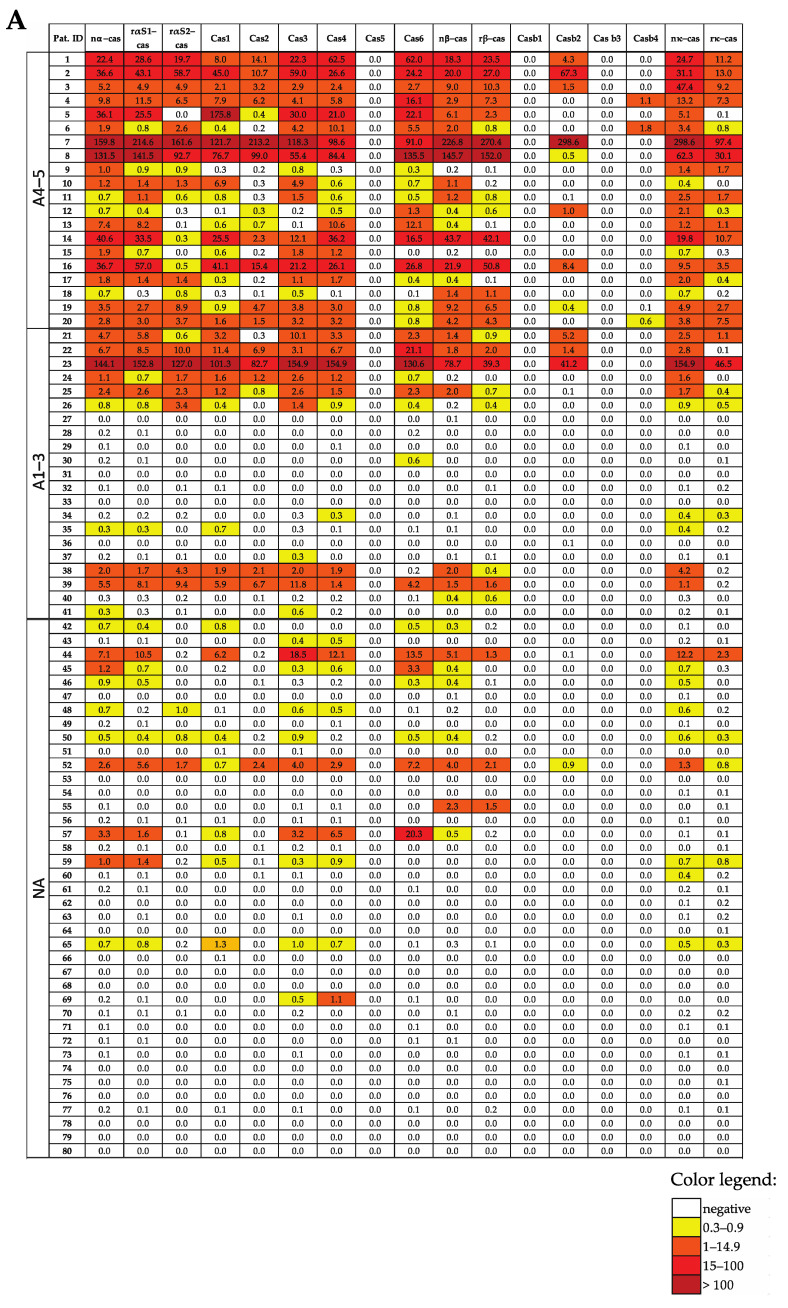

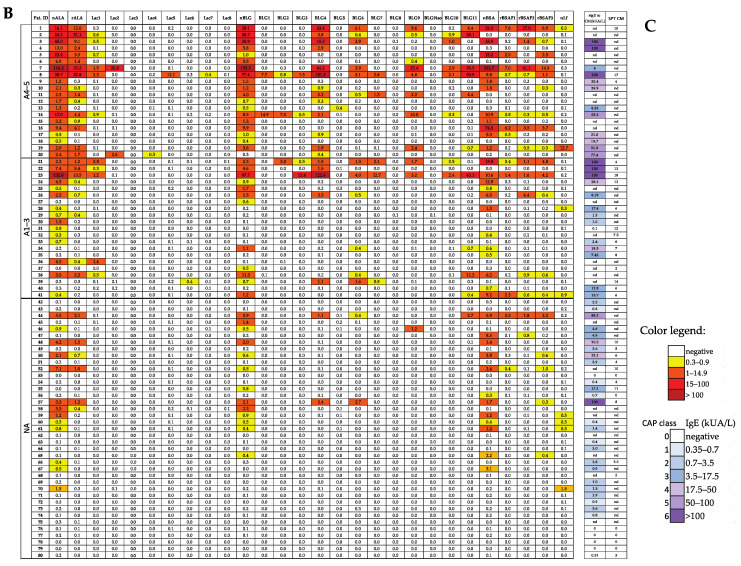
Heat map of the IgE levels specific for caseins and casein-derived peptides (**A**), other milk allergen proteins and peptides thereof (**B**), and milk allergen extract-specific IgE levels and SPT results (**C**) in patients suffering from different intensities of CMA (A4–5, milk-related anaphylaxis with a Sampson grade of 4–5; A1–3, milk-related anaphylaxis with a Sampson grade of 1–3; and NA, without anaphylaxis). The IgE (ISU-E, kUA/L) levels are shown with a color code.

Figure 2A,B show the levels of IgE that are specific for CM allergens, recombinant BSA fragments and CM-allergen-derived peptides in the study population with and without CM-related anaphylaxis (Figure 2A,B; Table 1). We found that each of the patients with anaphylactic reactions to CM, in Sampson grades of 1–5 (*n* = 41), showed an IgE reactivity to at least one of the CM-allergen components. The heat map indicates already that patients with Sampson grades of 4–5 exhibited higher specific IgE levels than patients with anaphylaxis grades of 1–3 (Figure 2A,B).

In total, 17 out of 41 patients with anaphylactic reactions to CM had been tested via SPT for CM allergen extracts (Table 1). Moreover, 1 of the 17 patients with grades 1–3 tested negative via skin testing with CM allergen extracts (Table 1). Furthermore, 26 of the 41 patients with anaphylactic reactions and CM-allergen-specific IgE levels to CM allergen extracts were assessed via ImmunoCAP testing. One of the patients (i.e., patients #31) with an anaphylactic reaction of grade 1–3 showed CM-allergen-extract-specific IgE levels 0.12 kUA/L, which is CAP class 0, according to the established cut-off 0.35 kUA/L, despite the fact that the technical cut-off for ImmunoCAP is 0.1 kUA/L. Thus, 2 out of 29 patients who had been tested with conventional allergen-extract-based methods (i.e., either SPT and/or ImmunoCAP) tested negative, whereas CM-allergen-specific IgE levels could be detected in all 41 patients with anaphylactic reactions to CM with the use of a MAMA.

### 3.4. All CM-Allergic Patients with a Sampson Grade of 4–5 (but Bot Those with a Score of 1–3) Show Casein-Specific IgE Reactivity

When comparing the IgE reactivity profiles and the levels of patients with anaphylactic reactions with CM grades of 1–3 with the patients who had grade 4–5 reactions, we found that all patients who had grade 4–5 reactions showed IgE reactivity to at least one of the caseins (particularly alpha-casein (Figure 2A)). By contrast, only 12 out of 21 grade 1–3 patients showed IgE reactivity to caseins, 10 to alpha-casein, 1 to beta-casein (i.e., patient 40) and 1 to kappa-casein (i.e., patient 34) (Figure 2A). Interestingly, patient 34 showed IgE levels above the cut-off threshold (i.e., 0.3 ISU) of the alpha-casein-derived peptide Cas4 but not of the complete alpha-casein molecule (Figure 2A).

The nine patients with a grade of 1–3 who were negative to caseins showed IgE reactivity to alpha-lactalbumin (*n* = 7) or beta-lactoglobulin (*n* = 2). Of note, all seven alpha-lactalbumin-positive patients were negative to caseins and beta-lactoglobulin but showed grade 1–3 reactions. However, BSA was also recognized in casein-negative patients, but these patients were positive to alpha-lactalbumin and/or beta-lactoglobulin (Figure 2A,B).

### 3.5. The Use of MAMA Reveals IgE Reactivity to Cryptic Milk-Allergen-Derived Peptides

We made an interesting observation when comparing the IgE reactivities to the complete allergens and allergen-derived peptides in our study population. In general, IgE reactivity and specific IgE levels to allergen-derived peptides were lower than those for complete allergens. However, we found six patients who showed IgE reactivity to cryptic allergen-derived peptides without exhibiting allergen-specific IgE reactivity. Patients #30, #34, #37 and #69 showed IgE reactivity to alpha-casein-derived peptides but not to alpha-casein (Figure 2A). Furthermore, patient #39 showed IgE reactivity to the alpha-lactalbumin peptide Lac6 but not to the complete allergen, and patient #20 showed IgE levels against the beta-lactoglobulin peptide BLG4 but not against beta-lactoglobulin (Figure 2B).

### 3.6. IgE Levels to CM Allergens and Allergen-Derived Peptides Are Higher in Patients with Anaphylactic Reactions to CM than in Patients without Anaphylactic Symptoms

**Figure 3:**
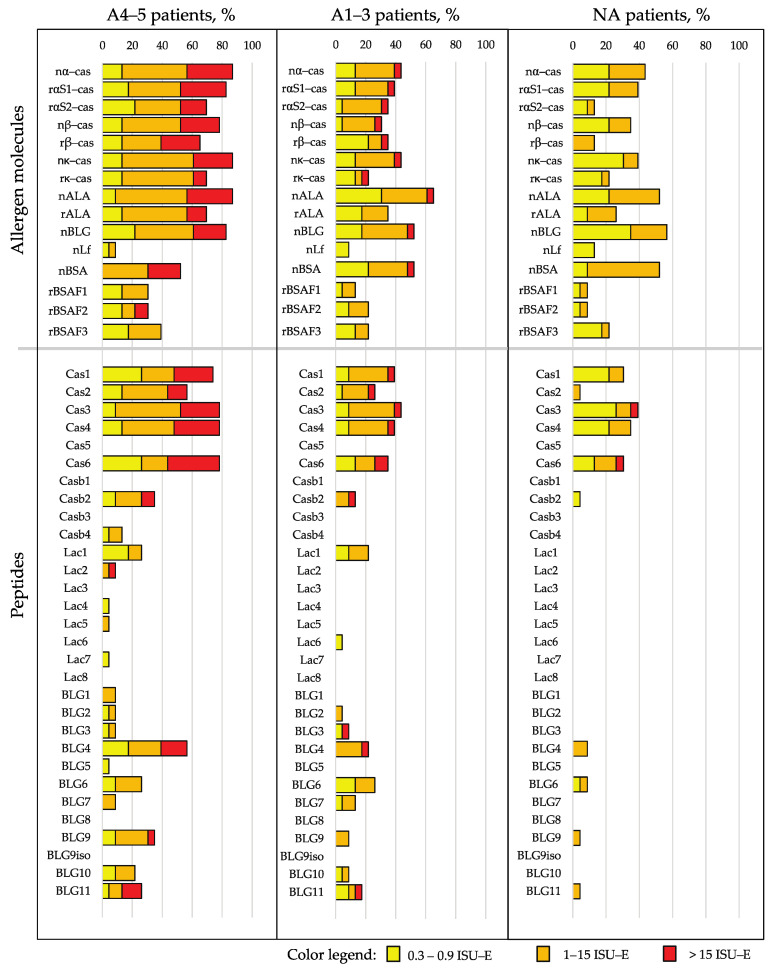
Frequencies and intensities of IgE recognition (the percentages of reactive sera and IgE levels according to a color code) of milk allergen proteins and peptides in patients with anaphylaxis according to Sampson criteria of 4–5 (A4–5 patients), with anaphylaxis according to Sampson criteria of 1–3 (A1–3 patients) and without anaphylaxis (NA patients).

**Figure 4:**
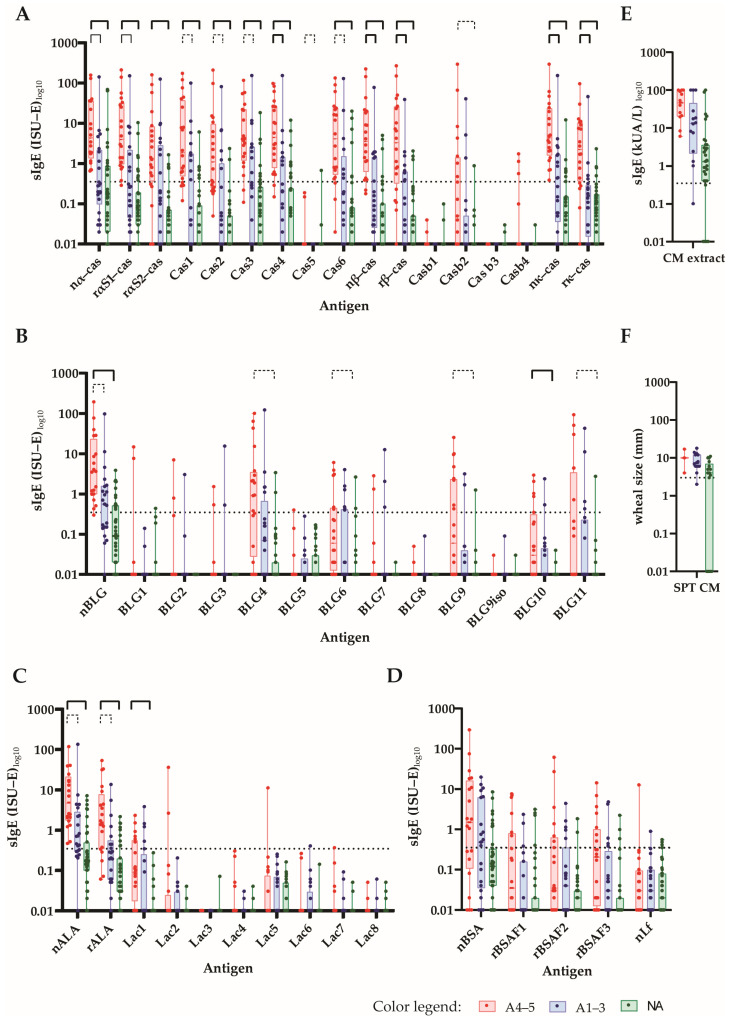
The IgE levels of CM-allergic patients to micro-arrayed milk proteins and peptides. The specific IgE levels (*y*-axes: ISU-E or kUA/L log10, box and whisker diagrams showing the first and third quartiles, as well as the minima and maxima, i.e., whiskers) toward (**A**) caseins and casein-derived peptides (**B**); β-lactoglobulins and β-lactoglobulin-derived peptides (**C**); α-lactalbumin and α-lactalbumin-derived peptides (**D**); BSA recombinant BSA fragments and lactoferrin, (**E**) cow’s milk allergen extract and (**F**) SPT results (mean wheal diameters in mm) in patients with a Sampson grade of 4–5 (A4–5: red), a Sampson grade of 1–3 (A1–3: blue) and those without anaphylaxis (NA: green) are shown. Statistically significant differences between the groups are shown by a capped line as in the above, *p*-value << 0.001 (Holm-corrected to 0.000385; continuous bold line), *p*-value < 0.001 (continuous line) and *p*-value < 0.01 (dotted line).

Figure 3 shows, in patients with and without anaphylactic reactions, the frequencies of IgE reactivity, as well as the specific IgE levels for the individual CM allergens and CM-allergen-derived peptides. The frequency of the IgE recognition of caseins and casein-derived peptides was approximately twice as high in patients with a grade of 4–5 when compared to patients with a grade of 1–3 and those without anaphylactic reactions (Figure 3). Importantly, grade 4–5 patients showed higher IgE levels to caseins and casein-derived peptides than grade 1–3 patients and those patients without anaphylaxis (Figure 3 and Figure 4A).

### 3.7. CM-Allergic Patients Who Develop Tolerance after an Elimination Diet Show a Drop of Allergen-Specific IgE When Using MAMA

**Figure 5:**
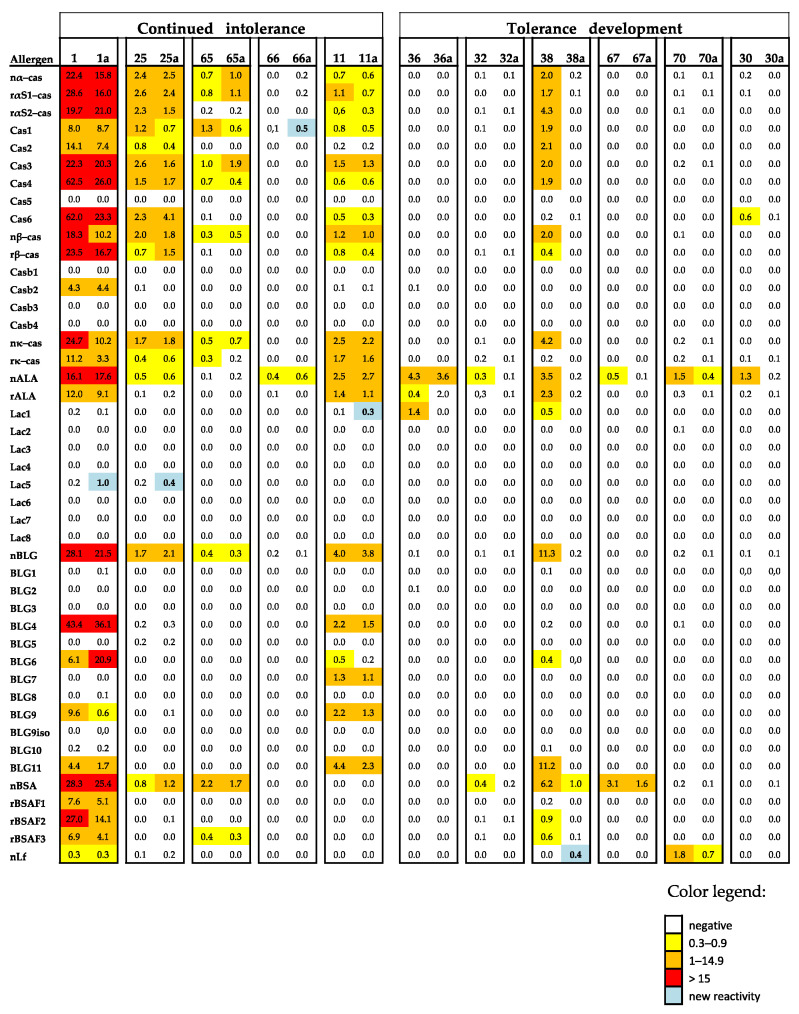
Heat map of the IgE levels specific for caseins and casein-derived peptides and other milk allergen proteins and peptides thereof in the sera of patients with continued intolerance and those developing tolerance before and after elimination (a). The IgE (ISU-E) levels are shown with a color code.

For 11 of the patients (Table 2), 7 were with and 4 were without anaphylactic reactions to CM. We had the chance to compare the allergen- and peptide-specific IgE levels before and after an elimination diet with the use of a MAMA (Table 2, Figure 5). A total of six of the patients developed partial tolerance to CM because they were able to eat baked or fermented milk products after an elimination diet (Table 2). The patients who developed tolerances were the grade 1–3 patients or those patients without CM-related anaphylactic reactions. Each of the six patients who developed tolerance showed a decline in allergen- and/or peptide-specific IgE, whereas no relevant decline in specific IgE levels was noted for the five patients who did not develop tolerance (Figure 5). Further, two out of the five patients who did not develop tolerance were grade 4–5 patients. No relevant differences regarding the duration of the time of the elimination diet were noted among the tolerant or intolerant patients (Table 2).

## 4. Discussion

Different mechanisms may be responsible for CM intolerance, and among them are lactose intolerance, IgE-mediated allergy and others [3]. In order to confirm the diagnosis of an IgE-mediated allergy, the detection of CM-allergen-specific IgE antibodies is crucial [4,5]. Serological tests for the detection of CM-allergen-specific IgE antibodies based on allergen extracts do not allow one to discriminate between the molecular IgE sensitization profiles [13]. For example, IgE sensitization to BSA is usually not associated with severe allergic reactions to CM exposure and can be a result of primary respiratory sensitization to albumins from pets [18]. On the other hand, IgE sensitization to caseins, beta-lactoglobulin and alpha-lactalbumin is often associated with anaphylactic symptoms [13]. Accordingly, multi-allergen tests containing different milk allergen molecules or CM-allergen-derived peptides have been developed [19,20,21,22]. These tests have been useful methods through which to establish molecular IgE sensitization profiles, monitor the effects of CM AIT and establish the development of tolerance to CM [21,22,23,24,25].

The milk allergen micro-array (MAMA) developed by us is unique and different from published and currently available multi-allergen tests for measuring CM-specific IgE antibodies (which either contain only purified CM allergen molecules [19] or only peptides and fragments from certain cow’s milk allergens [20,21,22]). By contrast, the MAMA used in this study contains each of the milk allergens (i.e., caseins, alpha-lactalbumin, beta-lactoglobulin, BSA and lactoferrin), as well as the CM-allergen-derived peptides. According to the results obtained in our cohort of patients with anaphylactic symptoms to CM, all patients with IgE-mediated anaphylactic reactions were identified through the use of the MAMA, whereas this was not the case when using established methods for the quantification of CM-allergen-specific IgE (i.e., ImmunoCAP) or when in vivo testing (i.e., SPT) with CM allergen extracts was performed. Thus, the use of the MAMA appears to be superior to the latter allergen-extract-based methods for detecting IgE-mediated sensitization in CM-specific anaphylactic patients. However, although there was a good concordance between IgE positivity measured by MAMA and allergen extract-based ImmunoCAP and SPT, concordance was not complete for all children with non-anaphylactic reactions to cow’s milk (Figure 2). Similar results have been reported by other authors for micro-array-based diagnostics [35,36]. Another limitation of our study was that we have not tested sera from non-allergic subjects with MAMA. Yet, there are advantages of using MAMA over traditional ImmunoCAP detection methods of CM-allergen-specific IgE: With the use of a MAMA, IgE sensitization to multiple allergens and allergen-derived peptides can be detected with an incredibly small amount of serum, whereas almost twice the volume is needed for one ImmunoCAP determination. This advantage is particularly important for measuring specific IgE sensitization in small children from whom it may be difficult to obtain large amounts of serum. When using MAMA, a capillary blood sample is sufficient. It should be also mentioned that we found an excellent correlation of allergen-specific IgE levels determined by MAMA and the approved ImmunoCAP ISAC micro-array (Figure S1), although some discordances were noted which may be attributed to some differences regarding technology and allergen preparations.

Yet another important aspect of MAMA should be mentioned, which may become more relevant if MAMA is further refined for routine diagnosis. In fact, the production costs of MAMA are relatively low; thus, it may be possible to provide MAMA for the large-scale screening of IgE sensitization to milk in children.

The testing of IgE reactivity profiles to allergen molecules and allergen-derived peptides with MAMA revealed certain interesting and notable findings. For example, we found that certain CM-allergic children reacted only to allergen-derived peptides, so-called “cryptic epitopes”, which seem to be hidden in the intact allergen molecules and may become exposed only after digestion and/or the denaturation of the allergens. On the other hand, many CM-allergic patients suffering from CM-related anaphylaxis reacted only with intact allergen molecules; thus, this diagnosis would have escaped the diagnostic tests that are based only on allergen-derived peptides [20,21,22].

Furthermore, the use of MAMA appeared to be useful for monitoring the development of CM tolerance following an elimination diet, although tolerance was not assessed by controlled challenges in the clinic. In fact, we found that those patients who developed some form of CM tolerance upon the reintroduction of milk in the diet showed a reduction in allergen-specific IgE levels, whereas this was not observed for patients who did not develop signs of clinical tolerance.

In our study, we were not able to investigate patients who underwent AIT or OIT with CM allergens and therefore we do not have data regarding the development of allergen-specific IgG. However, the technology used by us for generating the MAMA is based on the well-established printing of allergens or peptides on pre-activated glass slides, which has been shown to also be useful for measuring allergen-specific IgG (IgG_1_, IgG_4_) responses [30]. Although not investigated in this study, it is known that under the given amounts of immobilized antigens in MAMA, one would expect that the blocking effect of AIT-induced IgG can be visualized via the competition with IgE binding by blocking the IgG antibodies that can be visualized by a reduction in allergen-specific IgE binding as a surrogate biomarker for the effects of AIT [30,37,38].

Although we were able to test a relatively large number of sera from CM-allergic children from different centers, it may be considered a limitation of our study that no double-blind, placebo-controlled food challenges had been performed in our patients. However, clinicians were able to provide a meticulous clinical characterization of the milk-allergic children, and this corresponded with the requirements set by international guidelines [26], including the presence of the clinical symptoms of immediate types of CMA that could be unambiguously attributed to CM and that had a clear-cut history of anaphylaxis following milk consumption [27].

In conclusion, we developed MAMA, containing milk allergens and allergen-derived peptides, as a useful research tool for the detection of IgE sensitization to multiple allergen molecules and allergen-derived peptides in CM-allergic patients, particularly for children that requires only small amounts of serum samples. 

## Figures and Tables

**Table 1:** Demographic, clinical and serological characterizations of CM-allergic patients.

Patients	Number	Gender	Age	CM-Related Symptoms	SPT CM	Total IgE	sIgE to CM	Other Allergy
	*n* % of total	f/m (%)	Years [Q_1_–Q_3_] (min–max)	Type: *n*	*n*	kU/L [Q_1_–Q_3_] (min–max), *n*	kUA/L [Q_1_–Q_3_] (min-max), *n*	Type: *n*
Total CM	80	28/52	MV: 3.1	Skin: 62	pos: 27	MV: 99	MV: 4.6	BA: 18
allergic	100%	(35/65%)	[2.0–5.5]	GI: 42	neg: 6	[34.1–649]	[0.9–24.3]	AR: 28
			(0.5–12)	Sys: 41	nd: 46	(6.7–7372) *n* = 67	(0–100) *n* = 57	FA: 52
With anaphylaxis	41	14/27	MV: 5.0	Skin: 29	pos: 16	MV: 212	MV: 20.4	BA: 12
	51.25%	(34.2/65.8%)	[2.9–8.5]	GI: 20	neg: 1	[45.9–705]	[8–93.9]	AR: 18
			(1–12)	Sys: 41	nd: 24	(6.7–7372) *n* = 36	(0.12–100) *n* = 26	FA: 29
A4-5	20	7/13	MV: 4.9	Skin: 14	pos: 3	MV: 257.5	MV: 46.6	BA: 8
Sampson score 4-5	25%	(35/65%)	[2.9–8.8]	GI: 9	neg: 0	[95–791.3]	[20–98]	AR: 9
			(2.3–12)	Sys: 20	nd: 17	(6.7–7372) *n* = 18	(6–100) *n* = 12	FA: 15
A1-3	21	7/14	MV: 5.0	Skin: 15	pos: 13	MV: 95	MV: 13.3	BA: 4
Sampson score 1-3	26.25%	(33.3/66.7%)	[2.5–8.5]	GI: 11	neg: 1	[43.3–699.3]	[2.1–46.1]	AR: 9
			(1–11.2)	Sys: 21	nd: 7	(20.2–4030) *n* = 18	(0.12–100) *n* = 14	FA: 14
NA	39	14/25	MV: 2.5	Skin: 33	pos: 11	MV: 68.5	MV: 1.3	BA: 6
Without	48.75%	(35.9/64.1%)	[1.3–3.9]	GI: 22	neg: 5	[23.3–311]	[0.4–3.6]	AR: 10
anaphylaxis			(0.5–6)	Sys: 0	nd: 22	(7.5–1661) *n* = 31	(0–100) *n* = 31	FA: 23

Abbreviations: f, female; m, male; CM, cow’s milk; *n*, numbers; MV, median value; Skin, skin symptoms; GI, gastrointestinal symptoms; Sys, systemic reactions; SPT, skin prick test; BA, bronchial asthma; AR, allergic rhinitis; FA, food allergy; nd, not done.

**Table 2:** Demographic, clinical and immunological characterizations of CM-allergic patients who underwent CM diet for different durations.

			Time							
Patient ID	Gender	Age (Months)	Difference (Months)	Tolerance Development	Tolerance to Milk	Symptoms		Eosinophils		Total IgE
						Severity	Other	%	×10^9^/L	(kU/L)
1	f	34	18		no	5	BA, AR, GI	8	0.68	440
1a		52		no	no	5	BA, AR, GI	5	0.6	440
25	m	44	18		no	3	AD, BA	5	0.4	39.5
25a		62		no	no	3	AD, BA	5	0.4	39.5
65	m	72	9		baked	0	AD, AR, GI	6.3	0.42	nd
65a		81		no	baked	0	AD, AR, GI	6.3	0.42	nd
66	m	16	18		baked	0	AD, AR, GI	2	0.02	248.7
66a		34		no	baked	0	AD, AR, GI	2	0.13	127.8
11	m	58	11		no	4	AD, AR, GI	8	0.67	nd
11a		69		no	no	4	AD, AR, GI	5	0.4	nd
36	f	35	18		no	2	AR, GI	3.6	0.3	32.3
36a		53		yes	baked	0	AR	3.6	0.3	32.3
32	m	134	9		no	2	AR, GI	3.7	0.3	48.1
32a		143		yes	baked	0	0	nd	nd	nd
38	f	88	18		no	2	AD, AR, GI	3	0.2	82.5
38a		106		yes	baked	0	0	nd	nd	nd
67	m	30	18		baked	0	AD, GI	4	0.24	78.8
67a		48		yes	fermented	0	AD	4.6	0.3	58.6
70	m	67	12		baked	0	AD, BA, AR, GI	8.8	1	nd
70a		79		yes	fermented	0	BA, AR	11.6	1.27	nd
30	m	21	8		no	3	AD, GI	8	0.68	nd
30a		29		yes	baked	0	AD	3.2	0.3	nd

Abbreviations: f, female; m, male; CM, cow’s milk; GI, gastrointestinal symptoms; BA, bronchial asthma; AR, allergic rhinitis; AD, atopic dermatitis; nd, not done.

## Data Availability

All data are available upon reasonable request. Furthermore, the MAMA will be made available as a research tool upon reasonable request for others to use in the course of collaborative research projects.

## Supplementary Materials

The following supporting information can be downloaded at https://www.mdpi.com/article/10.3390/nu15102401/s1, Table S1: Sources, sequences and characteristics of the allergens and peptides of MAMA; Table S2: Detailed statistical analysis of the differences in median IgE levels specific for CM allergens and CM-allergen-derived peptides in patients with anaphylaxis according Sampson criteria 4–5 (A4–5 patients), with anaphylaxis according to Sampson criteria 1–3 (A1–3 patients) and those without anaphylaxis (NA patients); Figure S1: Heat map (A) and correlation (B) of IgE levels (ISU-E) specific for cow’s milk allergen molecules (na-cas, nAla, nBLG, nBSA) as determined by MAMA (x-axes) versus ImmunoCAP ISAC (y-axes).

## Abbreviations

MAMA	Milk allergen micro-array
CM	Cow´s milk
CMA	Cow’s milk allergy
BSA	Bovine serum albumin
HSA	Human serum albumin
AIT	Allergen-specific immunotherapy
OIT	Oral immunotherapy
GI	Gastrointestinal symptoms
BA	Bronchial asthma
AR	Allergic rhinitis
AD	Atopic dermatitis
FA	Food allergy
SPT	Skin prick test
MV	Median value
ISU	ISAC standardized units
ISU-E	IgE level presented in ISAC standardized units
A4–5	Patients with milk-related anaphylaxis with a Sampson score of 4–5
A1–3	Patients with milk-related anaphylaxis with a Sampson score of 1–3
NA	Patients without anaphylaxis
na-cas	natural alpha casein
nALA	natural alpha lactalbumin
nBLG	natural betalagtoglobulin
