# Supplementary material for: Milk Allergen Micro-Array (MAMA) for Refined Detection of Cow’s-Milk-Specific IgE Sensitization
Source: Nutrients. 2023 May 21;15(10):2401. doi: 10.3390/nu15102401 (PMC10223406; doi:10.3390/nu15102401)
Supplement: Supplementary file 1 [file nutrients-15-02401-s001.zip › nutrients-2379059-supplementary.pdf]

nutrients

MDPI

Supplementary materials

Table S1. Sources, sequences and characteristics of allergens and peptides of MAMA.

Name	Proteins	
**nα-cas**	α-casein from bovine milk	Sigma (C6780)
**nβ-cas**	β-casein from bovine milk	Sigma (C6905)
**nκ-cas**	κ-casein from bovine milk	Sigma (C0406)
**nALA**	α-lactalbumin from bovine milk	Sigma (L5385)
**nBLG**	β-lactoglobulin from bovine milk	Sigma (L3908)
**nBSA**	Bovine serum albumin	Sigma (A2058)
**nLf**	Lactoferrin from bovine milk	Sigma (L9507)
**rαS1-cas**	MRPKHPIKHQ GLPQEVLNEN LLRFFVAPFP EVFGKEKVNE LSKDIGSEST EDQAMEDIKQMEAESISSSE EIVPNSVEQK HIQKEDVPSE RYLGYLEQLL RLKKYKVPQL EIVPNSAEERLHSMKEGIHA QQKEPMIGVN QELAYFYPEL FRQFYQLDAY PSGAWYYVPL GTQYTDAPSFSDIPNPIGSE NSEKTTMPLW **HHHHHH**	GenBank: EU221551.1 and ABW98936.1; *E. coli* BL21-expressed; [33]
**rαS2-cas**	MKNTMEHVSS SEESIISQET YKQEKNMAIN PSKENLCSTF CKEVVRNANEEEYSIGSSSE ESEAVATEEV KITVDDKHYQ KALNEINQFY QKFPQYLQYLYQGPIVLNPW DQVKRNAVPI TPTLNREQLS TSEENSKKTV DMESTEVFTKKTKLTEEEKN RLNFLKKISQ RYQKFALPQY LKTVYQHQKA MKPWIQPKTK VIPYVRYL **HHHHHH**	*E. coli* BL21-expressed; [9]
**rβ-cas**	MRELEELNVP GEIVESLSSS EESITRINKK IEKFQSEEQQ QTEDELQDKI HPFAQTQSLV YPFPGPIPNS LPQNIPPLTQ TPVVVPPFLQ PEVMGVSKVK EAMAPKHKEM PFPKYPVEPF TESQSLTLTD VENLHLPLPL LQSWMHQPHQ PLPPTVMFPP QSVLSLSQSK VLPVPQKAVP YPQRDMPIQA FLLYQEPVLG PVRGPFPIIV **HHHHHH**	*E. coli* BL21-expressed; [9]
**rκ-cas**	MQEQNQEQPI RCEKDERFFS DKIAKYIPIQ YVLSRYPSYG LNYYQQKPVA LINNQFLPYP YYAKPAAVRS PAQILQWQVL SNTVPAKSCQ AQPTTMARHP HPHLSFMAIP PKKNQDKTEI PTINTIASGE PTSTPTIEAV ESTVATLEAS PEVIESPPEI NTVQVTSTAV **HHHHHH**	*E. coli* BL21-expressed; [9]
**rALA**	MEQLTKCEVF RELKDLKGYG GVSLPEWVCT TFHTSGYDTQ AIVQNNDSTE YGLFQINNKI WCKDDQNPHS SNICNISCDK FLDDDLTDDI MCVKKILDKV GINYWLAHKA LCSEKLDQWL CEKL **HHHHHH**	NCBI Reference Sequence: NP_776803.1 (mature protein w/o leader sequence), *E. coli* BL21-expressed; [34]
**rBSAF1**	MRGVFRRDTH KSEIAHRFKD LGEEHFKGLV LIAFSQYLQQ CPFDEHVKLV NELTEFAKTC VADESHAGCE KSLHTLFGDE LCKVASLRET YGDMADCCEKQEPERNECFL SHKDDSPDLP KLKPDPNTLC DEFKADEKKF WGKYLYEIAR RHPYFYAPEL LYYANKYNGV FQECCQAEDK GACLLPKIET MREKVLTSS **HHHHHH**	AA 1 – 199 [GenBank: AAA51411.1]
**rBSAF2**	MARQRLRCAS IQKFGERALK AWSVARLSQK FPKAEFVEVT KLVTDLTKVH KECCHGDLLE CADDRADLAK YICDNQDTIS SKLKECCDKP LLEKSHCIAE	AA 200 – 389 [GenBank: AAA51411.1]

	VEKDAIPENL PPLTADFAED KDVCKNYQEA KDAFLGSFLY EYSRRHPEYA VSVLLRLAKE YEATLEECCA KDDPHACYST VFDKLKHLVDE **HHHHHH**	
**rBSAF3**	MPQNLIKQNC DQFEKLGEYG FQNALIVRYT RKVPQVSTPT LVEVSRSLGK VGTRCCTKPE SERMPCTEDY LSLILNRLCV LHEKTPVSEK VTKCCTESLV NRRPCFSALT PDETYVPKAF DEKLFTFHAD ICTLPDTEKQ IKKQTALVEL LKHKPKATEE QLKTVMENFV AFVDKCCAAD DKEACFAVEG PKLVVSTQTA LA **HHHHHH**	AA 390 – 591 [GenBank: AAA51411.1]
**Name**	**Peptides**	
**Cas1**	RPKHPIKHQG LPQEVLNENL LRFFVAPFPE V**C**	AA 2 - 32 + 1 Cys [33]
**Cas2**	FGKEKVNELS KDIGSESTED QAMEDIKQME AES**C**	AA 33 - 65 + 1 Cys [33]
**Cas3**	ISSSEEIVPN SVEQKHIQKE DVPSERYLGY EQLLR**C**	AA 34 - 95, 97-101 + 1 Cys [33]
**Cas4**	CLKKYKVPQL EIVPNSAEER LHSMKEGIHA QQKE	1 Cys + AA 102 – 134 [33]
**Cas5**	CPMIGVNQEL AYFYPELFRQ FYQLDAYPSG AWYYV	1 Cys + AA 135 – 168 [33]
**Cas6**	PLGTQYTDAP SFSDIPNPIG SENSEKTTMP LW**C**	AA 169 – 200 + 1 Cys [33]
**Casb1**	HQPHQPLPPT V	AA 146 – 156 [GenBank: XP_005902099.2]
**Casb2**	VYPFPGPIPN	AA 60 – 69 [GenBank: XP_005902099.2]
**Casb3**	LSSSEE	AA 17 – 21 [GenBank: XP_005902099.2]
**Casb4**	PVVVPPFL	AA 82 – 89 [GenBank: XP_005902099.2]
**Lac1**	EQLTKCEVFR ELKDLKGYG	AA 2 – 20 [34]
**Lac2**	LKGYGGVSLP EWVCTTFHT S	AA 16 – 35 [34]
**Lac3**	TFHTSGYDTQ AIVQNNDSTE	AA 31 – 50 [34]
**Lac4**	NDSTEYGLFQ INNKIWCKDD	AA 46 – 65 [34]
**Lac5**	WCKDDQNPHS SNICNISCDK	AA 61 – 80 [34]
**Lac6**	ISCDKFLDDD LTDDIMCVKK	AA 76 – 95 [34]
**Lac7**	MCVKKILDKV GINYWLAHKA	AA 91 – 110 [34]
**Lac8**	LAHKALCSEK LDQWLCEKL	AA 106 – 124 [34]
**BLG1**	LIVTQTMKGL DIQKVA	AA 2 – 17 [GenBank: CAA32835.1]
**BLG2**	IQKVAGTWYS LAMAASDISL	AA 13 – 32 [GenBank: CAA32835.1]
**BLG3**	SDISLLDAQS APLRVYVEEL	AA 28 – 47 [GenBank: CAA32835.1]
**BLG4**	YVEELKPTPE GDLEILLQKW	AA 43 – 62 [GenBank: CAA32835.1]

**BLG5**	LLQKWENGEC AQKKIIAEKT	AA 58 – 77 [GenBank: CAA32835.1]
**BLG6**	IAEKTKIPAV FKIDALNENK	AA 73 – 92 [GenBank: CAA32835.1]
**BLG7**	LNENKVLVLD TDYKKYLLFC	AA 88 – 107 [GenBank: CAA32835.1]
**BLG8**	YLLFCMENSA EPEQSLACQC	AA 103 – 122 [GenBank: CAA32835.1]
**BLG9**	LACQCLVRTP EVDDEALEKF	AA 118 – 137 [GenBank: CAA32835.1]
**BLG9iso**	LVCQCLVRTP EVDDEALEKF	AA 123 – 142 [GenBank: CAA32835.1]
**BLG10**	ALEKFDKALK ALPMHIRLSF	AA 133 – 152 [GenBank: CAA32835.1]
**BLG11**	IRLSFNPTQL EEQCHI	AA 148 – 163 [GenBank: CAA32835.1]

References :

33. Schulmeister, U.; Hochwallner, H.; et al. Cloning, expression, and mapping of allergenic determinants of alphaS1-casein, a major cow's milk allergen. *J Immunol*. 2009 Jun 1;182(11):7019-29. doi: 10.4049/jimmunol.0712366.

9. Hochwallner, H.; Schulmeister, U.; Swoboda, I.; Balic, N.; Geller, B.; Nystrand, M.; Härlin, A.; Thalhamer, J.; Scheiblhofer, S.; Niggemann, B.; Quirce, S.; Ebner, C.; Mari, A.; Pauli, G.; Herz, U.; van Tol, E.A.; Valenta, R.; Spitzauer, S. Microarray and allergenic activity assessment of milk allergens. *Clin Exp Allergy*. 2010 Dec;40(12):1809-18. doi: 10.1111/j.1365-2222.2010.03602.x.

34. Hochwallner, H.; Schulmeister, U.; et al. Visualization of clustered IgE epitopes on alpha-lactalbumin. *J Allergy Clin Immunol*. 2010 Jun;125(6):1279-1285.e9. doi: 10.1016/j.jaci.2010.03.007.

Table S2. Detailed statistical analysis of differences of median IgE levels specific for CM allergens and CM allergen-derived peptides in patients with anaphylaxis according Sampson's criteria 4-5 (A4-5 patients), with anaphylaxis according to Sampson's criteria 1-3 (A1-3 patients) and without anaphylaxis (NA patients). Highly significant differences (p-value <0.01) are indicated in red.

Allergen	Without anaphylaxis (n=20)	Sampson 1-3 (n=21)	Sampson 4-5 (n=20)	p-value (<0.01)			p-value (<0.001)		
				A1-3 vs A4-5	A1-3 vs NA	A 4-5 vs NA	A1-3 vs A4-5	A1-3 vs NA	A4-5 vs NA
nα-cas	0.214 [0.117; 0.995]	0.281 [0.092; 2.168]	4.342 [1.324; 36.483]	.000	.602	.000	.000	.602	.000
rαS1-cas	0.106 [0.052; 0.676]	0.260 [0.043; 2.169]	3.956 [0.945; 32.265]	.000	.434	.000	.000	.434	.000
rαS2-cas	0.027 [0.004; 0.136]	0.137 [0.012; 2.824]	1.346 [0.359; 8.271]	.032	.067	.000	.032	.067	.000
nβ-cas	0.067 [0.012; 0.369]	0.086 [0.024; 1.428]	3.546 [0.609; 19.604]	.000	.657	.000	.000	.657	.000
rβ-cas	0.019 [0.005; 0.203]	0.035 [0.006; 0,632]	3.283 [0.312; 26.105]	.000	.473	.000	.000	.473	.000
nκ-cas	0.143 [0.055; 0.607]	0.267 [0.033; 1.664]	4.331 [1.562; 23.495]	.000	.611	.000	.000	.611	.000
rκ-cas	0.079 [0.035; 0.244]	0.107 [0.016; 0.282]	2.243 [0.36; 10.327]	.000	.990	.000	.000	.990	.000
nALA	0.359 [0.156; 2.978]	0.693 [0.294; 2.893]	4.743 [1.75; 21.812]	.001	.211	.000	.001	.211	.000
rALA	0.087 [0.036; 0.645]	0.274 [0.063; 0.565]	1.533 [0.337; 7.892]	.003	.155	.000	.003	.155	.000
nBLG	0.488 [0.116; 1.278]	0.565 [0.147; 1.596]	3.564 [0.983; 23.515]	.003	.584	.000	.003	.584	.000
nLf	0.020 [0.007; 0.178]	0.027 [0; 0.099]	0.027 [0.006; 0.097]	.743	.628	.882	.743	.628	.882
nBSA	0.301 [0.064; 1.595]	0.478 [0.036; 6.313]	1.489 [0.107; 16.36]	.206	.979	.185	.206	.979	.185
rBSAF1	0.000 [0; 0.054]	0.009 [0; 0.162]	0.038 [0; 0.802]	.192	.385	.059	.192	.385	.059
rBSAF2	0.029 [0.004; 0.069]	0.038 [0; 0.355]	0.039 [0.008; 0.632]	.386	.793	.232	.386	.793	.232
rBSAF3	0.012 [0; 0.151]	0.027 [0.005; 0.288]	0.202 [0.017; 1.015]	.200	.332	.029	.200	.332	.029
Cas1	0.047 [0.001; 0.5]	0.037 [0; 1.74]	1.862 [0.475; 37.174]	.002	.625	.000	.002	.625	.000
Cas2	0.029 [0.005; 0.113]	0.031 [0; 1.001]	1.104 [0.247; 9.616]	.001	.793	.000	.001	.793	.000
Cas3	0.156 [0.035; 0.527]	0.286 [0.003; 2.577]	3.935 [1.173; 22.042]	.002	.794	.000	.002	.794	.000
Cas4	0.077 [0.005; 0.556]	0.200 [0; 1.434]	4.460 [0.771; 26.506]	.000	.753	.000	.000	.753	.000
Cas5	0.000 [0; 0]	0.000 [0; 0]	0.000 [0; 0.009]	.003	.311	.016	.003	.311	.016

Cas6	0.027 [0.006; 0.488]	0.111 [0.011; 1.502]	4.132 [0.516; 23.649]	.001	.375	.000	.001	.375	.000
Casb1	0.004 [0.002; 0.005]	0.002 [0; 0.003]	0.003 [0.002; 0.004]	.111	.026	.507	.111	.026	.507
Casb2	0.004 [0.001; 0.01]	0.012 [0; 0.048]	0.047 [0.006; 1.41]	.039	.416	.002	.039	.416	.002
Casb3	0.000 [0; 0]	0.000 [0; 0.002]	0.000 [0; 0.004]	.823	.226	.196	.823	.226	.196
Casb4	0.001 [0; 0.004]	0.000 [0; 0.002]	0.001 [0; 0.012]	.420	.576	.719	.420	.576	.719
Lac1	0.000 [0; 0.017]	0.000 [0; 0.255]	0.094 [0.009; 0.567]	.143	.166	.001	.143	.166	.001
Lac2	0.000 [0; 0.001]	0.000 [0; 0.027]	0.002 [0; 0.024]	.250	.593	.069	.250	.593	.069
Lac3	0.000 [0; 0.002]	0.000 [0; 0.002]	0.000 [0; 0.002]	.933	.406	.464	.933	.406	.464
Lac4	0.004 [0; 0.014]	0.000 [0; 0.01]	0.000 [0; 0.012]	.899	.275	.321	.899	.275	.321
Lac5	0.040 [0; 0.07]	0.000 [0; 0.07]	0.000 [0; 0.072]	.656	.344	.103	.656	.344	.103
Lac6	0.000 [0; 0]	0.000 [0; 0.027]	0.000 [0; 0.004]	.206	.082	.657	.206	.082	.657
Lac7	0.000 [0; 0.022]	0.000 [0; 0.014]	0.000 [0; 0]	.255	.712	.182	.255	.712	.182
Lac8	0.000 [0; 0.013]	0.000 [0; 0.008]	0.000 [0; 0.012]	.859	1.000	.852	.859	1.000	.852
BLG1	0.000 [0; 0.004]	0.000 [0; 0.005]	0.000 [0; 0.01]	.493	.953	.548	.493	.953	.548
BLG2	0.001 [0; 0.003]	0.002 [0; 0.007]	0.004 [0; 0.007]	.744	.296	.109	.744	.296	.109
BLG3	0.000 [0; 0.001]	0.000 [0; 0.002]	0.001 [0; 0.006]	.521	.296	.103	.521	.296	.103
BLG4	0.020 [0.002; 0.092]	0.072 [0; 0.674]	0.922 [0.024; 3.544]	.020	.415	.003	.020	.415	.003
BLG5	0.021 [0; 0.076]	0.000 [0; 0.029]	0.000 [0; 0.008]	.275	.107	.018	.275	.107	.018
BLG6	0.004 [0; 0.026]	0.008 [0; 0.422]	0.059 [0.014; 0.46]	.053	1.000	.009	.053	1.000	.009
BLG7	0.000 [0; 0.009]	0.000 [0; 0.004]	0.000 [0; 0.011]	.697	.635	.557	.697	.635	.557
BLG8	0.000 [0; 0.001]	0.001 [0; 0.003]	0.001 [0; 0.011]	.874	.078	.122	.874	.078	.122
BLG9	0.000 [0; 0.003]	0.002 [0; 0.039]	0.058 [0; 2.366]	.044	.128	.002	.044	.128	.002
BLG9iso	0.000 [0; 0.001]	0.00 [0; 0]	0.000 [0; 0.003]	.525	.411	.899	.525	.411	.899
BLG10	0.003 [0.001; 0.005]	0.005 [0.002; 0.043]	0.032 [0.007; 0.312]	.060	.026	.000	.060	.026	.000
BLG11	0.000 [0; 0.01]	0.004 [0; 0.23]	0.006 [0; 3.465]	.336	.178	.027	.336	.178	.027

A

	MAMA	ISAC	MAMA	ISAC	MAMA	ISAC	MAMA	ISAC	MAMA	ISAC
**Pat. ID**	**nALA**	**nALA**	**nBLG**	**nBLG**	**nBSA**	**nBSA**	**nα-cas**	**nα-cas**	**nLf**	**nLf**
**1**	16.1	4.8	28.1	5.2	28.3	8.1	22.4	5.6	0.3	0.0
**2**	24.2	74.6	28.7	50.5	0.0	0.0	36.6	71.7	0.1	0.0
**3**	40.0	17.1	39.9	7.5	18.9	0.0	5.2	11.1	0.1	0.0
**4**	13.0	13.0	5.8	7.5	0.27	0.0	9.8	11.0	0.0	0.0
**5**	23.4	9.5	1.0	0.0	18.2	11.0	36.1	14.0	0.0	0.0
**6**	6.9	7.8	3.5	5.0	0.1	5.2	1.9	0.0	0.0	0.0
**7**	116.2	45.0	195.2	37.0	292.7	100.0	159.8	57.0	0.27	0.0
**8**	38.7	110.1	77.4	115.9	9.8	12.7	131.5	112.2	0.1	0.0
**9**	1.2	1.1	1.5	1.3	1.8	0.7	1.0	1.0	0.0	0.0
**10**	2.1	3.7	1.2	2.5	1.8	0.7	1.2	1.8	0.0	0.0
**11**	2.5	2.8	4.0	5.1	0.0	0.0	0.7	1.0	0.0	0.0
**12**	1.7	0.7	0.7	0.0	0.0	0.0	0.7	0.4	0.0	0.0
**13**	1.3	1.2	0.5	0.7	0.29	0.0	7.4	9.4	0.1	0.0
**14**	17.0	24.8	8.5	30.5	10.9	12.5	40.6	34.9	0.2	0.0
**15**	2.2	5.8	1.2	4.0	1.1	0.0	1.9	0.9	0.0	0.0
**16**	9.4	17.8	9.9	19.0	74.5	22.7	36.7	25.5	0.0	0.0
**17**	0.5	0.5	1.0	1.6	5.0	0.0	1.8	4.4	0.0	0.0
**18**	0.5	0.0	0.4	0.0	0.0	0.0	0.7	1.3	0.0	0.0
**19**	2.0	13.3	3.6	15.9	1.2	3.8	3.5	12.0	12.7	26.1
**20**	2.4	5.0	0.3	0.0	0.2	0.0	2.8	2.1	0.0	0.0
**21**	2.2	12.5	2.3	9.4	19.8	26.4	4.7	12.1	0.1	0.0
**22**	7.4	52.3	4.6	29.0	0.0	0.0	6.7	27.9	0.1	0.0
**23**	132.8	110.8	97.7	116.2	10.6	28.1	144.1	113.2	0.2	0.4
**24**	4.3	17.3	0.9	4.7	12.9	13.7	1.1	5.0	0.0	0.0
**25**	0.5	0.0	1.7	2.9	0.8	0.0	2.4	3.3	0.1	0.0
**26**	2.2	2.8	1.5	3.1	6.5	6.4	0.8	1.1	0.0	0.0
**27**	0.2	0.0	0.6	1.2	0.0	0.0	0.0	0.0	0.0	0.0
**28**	0.4	8.5	0.2	8.7	1.3	3.9	0.2	2.3	0.3	0.0
**29**	0.7	1.1	0.2	0.4	0.1	0.0	0.1	0.0	0.0	0.0
**30**	1.3	0.9	0.1	0.0	0.0	0.0	0.2	0.0	0.0	0.0
**31**	0.8	0.3	0.0	0.0	0.0	0.0	0.0	0.0	0.1	0.0
**32**	0.3	0.4	0.1	0.0	0.4	0.0	0.1	0.0	0.0	0.0
**33**	0.7	1.5	0.1	0.4	0.1	0.0	0.0	0.0	0.0	0.0
**34**	0.2	0.7	1.1	5.5	0.6	0.7	0.2	1.6	0.0	0.0
**35**	0.2	0.6	0.2	0.5	0.5	0.9	0.3	1.2	0.0	0.0
**36**	4.3	2.6	0.1	0.0	0.0	0.0	0.0	0.0	0.0	0.0
**37**	0.0	0.0	0.5	1.3	0.0	0.0	0.2	0.0	0.0	0.0
**38**	3.5	0.7	11.3	0.3	6.2	0.9	2.0	0.0	0.0	0.0
**39**	0.29	0.4	0.7	2.7	0.1	0.0	5.5	22.9	0.1	0.0
**40**	0.29	1.1	0.2	0.5	0.7	1.3	0.28	0.6	0.0	0.0
**41**	0.4	1.0	1.2	1.0	9.2	16.7	0.3	1.0	0.9	4.2
**42**	0.1	0.0	0.0	0.0	0.27	0.3	0.7	1.3	0.0	0.0
**43**	0.2	0.0	0.1	0.0	0.0	0.0	0.1	0.0	0.2	0.0
**44**	5.5	8.2	3.9	9.7	5.9	11.4	7.1	21.2	0.2	0.0
**45**	0.2	0.0	1.4	2.4	0.1	0.0	1.2	2.5	0.0	0.0
**46**	0.9	0.4	0.5	0.4	0.1	0.0	0.9	1.5	0.0	0.0
**47**	0.1	0.0	0.2	0.0	8.5	7.8	0.0	0.7	0.0	0.0
**48**	4.2	10.2	2.0	2.8	1.4	0.6	0.7	1.1	0.0	0.0
**49**	0.2	0.9	0.2	0.8	0.1	0.0	0.2	0.0	0.0	0.0
**50**	2.1	11.1	0.6	3.9	2.8	5.9	0.5	1.4	0.0	0.0
**51**	0.2	0.0	0.1	0.0	0.2	0.0	0.0	0.0	0.0	0.0
**52**	7.1	14.2	0.5	0.9	2.4	7.3	2.6	8.5	0.2	0.0
**53**	0.0	0.0	0.0	0.0	0.0	0.0	0.0	0.0	0.0	0.0
**54**	0.2	0.26	0.1	0.0	0.0	0.0	0.0	0.0	0.1	0.0
**55**	0.0	0.0	0.8	2.6	0.0	0.0	0.1	6.1	0.0	0.0
**56**	0.2	0.0	0.2	0.0	0.3	0.0	0.2	0.0	0.1	0.0
**57**	3.3	21.5	2.1	7.1	1.7	3.6	3.3	8.7	0.0	0.0
**58**	3.3	5.8	2.2	4.0	0.1	0.0	0.2	0.9	0.0	0.0
**59**	1.2	0.0	0.9	0.0	1.2	0.0	1.0	0.0	0.5	0.0
**60**	0.5	0.0	0.5	0.0	0.4	0.0	0.1	0.0	0.5	0.0
**61**	0.8	0.0	0.4	0.0	1.2	0.0	0.2	0.0	0.3	0.0
**62**	0.1	0.0	0.0	0.0	0.1	0.0	0.0	0.0	0.0	0.0
**63**	0.1	0.0	0.1	0.0	0.0	0.0	0.0	0.0	0.1	0.0
**64**	0.1	0.0	0.0	0.0	0.1	0.0	0.0	0.0	0.1	0.0
**65**	0.1	0.0	0.4	0.8	2.2	0.6	0.7	1.3	0.0	0.0
**66**	0.4	0.7	0.27	0.5	0.0	0.0	0.0	0.0	0.0	0.0
**67**	0.5	0.0	0.0	0.0	3.1	0.8	0.0	0.0	0.0	0.0
**68**	0.1	0.0	0.0	0.0	0.1	0.0	0.0	0.0	0.0	0.0
**69**	0.2	0.0	0.0	0.0	0.0	0.0	0.2	0.0	0.0	0.0
**70**	1.5	2.0	0.2	0.0	0.2	0.0	0.1	0.0	1.8	0.8
**71**	0.1	0.0	0.1	0.0	0.2	0.0	0.1	0.0	0.1	0.0
**72**	0.28	0.0	0.0	0.0	0.0	0.0	0.1	0.0	0.1	0.0
**73**	0.2	0.0	0.2	0.0	0.2	0.0	0.1	0.0	0.1	0.0
**74**	0.1	0.0	0.1	0.0	0.2	0.0	0.0	0.0	0.0	0.0
**75**	0.25	0.0	0.0	0.0	0.0	0.0	0.0	0.0	0.0	0.0
**76**	0.1	0.0	0.1	0.0	0.1	0.0	0.0	0.0	0.0	0.0
**77**	0.2	0.0	0.1	0.0	0.2	0.0	0.2	0.0	0.0	0.0
**78**	0.0	0.0	0.0	0.0	0.0	0.0	0.0	0.0	0.0	0.0
**79**	0.0	0.0	0.0	0.0	0.0	0.0	0.0	0.0	0.0	0.0
**80**	0.2	0.0	0.0	0.0	0.1	0.0	0.0	0.0	0.0	0.0

IgE (ISU-E)
- negative
- 0.3-0.9
- 1-14.9
- 15-100
- > 100

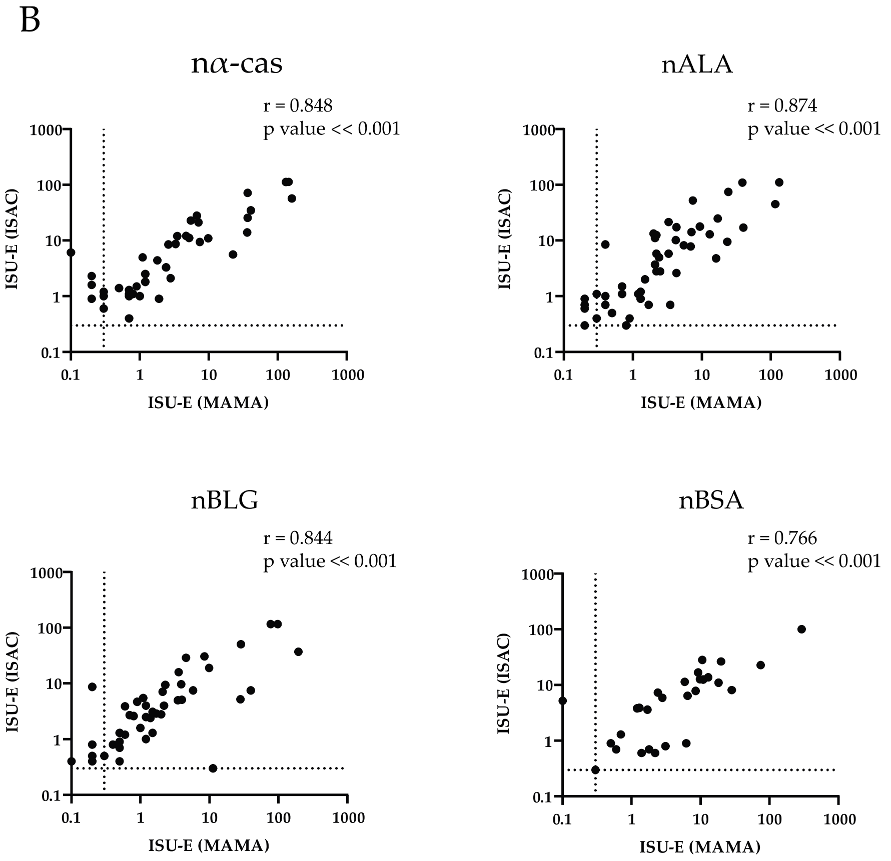

Figure S1. Heat map (**A**) and correlation (**B**) of IgE levels (ISU-E) specific for cow´s milk allergen molecules (nα-cas, nAla, nBLG, nBSA) as determined by MAMA (x-axes) versus ImmunoCAP ISAC (y-axes).
